# Primary healthcare provision and Chronic Fatigue Syndrome: a survey of patients' and General Practitioners' beliefs

**DOI:** 10.1186/1471-2296-6-49

**Published:** 2005-12-13

**Authors:** Marie A Thomas, Andrew P Smith

**Affiliations:** 1 Centre for Occupational and Health Psychology, School of Psychology, Cardiff University, 63 Park Place, Cardiff, UK

## Abstract

**Background:**

The current study was conducted as part of a research project into the evaluation and assessment of healthcare provision and education in Chronic Fatigue Syndrome (CFS). One aim of the study was the development of informative and educational literature for both General Practitioners (GP) and sufferers. Issues such as diagnosis, management and treatment of the syndrome should be included in information booklets written by healthcare professionals. It was important to begin the process by assessing the level of specialist knowledge that existed in typical GP surgeries. This data would then be compared to data from CFS patients.

**Method:**

197 survey booklets were sent to CFS sufferers from an existing research panel. The patients approached for the purpose of the study had been recruited onto the panel following diagnosis of their illness at a specialised CFS outpatient clinic in South Wales. A further 120 booklets were sent to GP surgeries in the Gwent Health Authority region in Wales.

**Results:**

Results from the study indicate that the level of specialist knowledge of CFS in primary care remains low. Only half the GP respondents believed that the condition actually exists.

**Conclusion:**

Steps are recommended to increase the knowledge base by compiling helpful and informative material for GPs and patient groups.

## Background

A patient with Chronic Fatigue Syndrome (CFS) is described as one suffering unrelenting, debilitating fatigue (for a period of six months or more) which is unresolved by rest. This fatigue is not the result of normal physical activity and can cause both mental and physical impairment to the sufferer. Furthermore, the fatigue experienced is not as a result of an ongoing medical condition. Chronic Fatigue Syndrome remains a poorly understood condition and still poses problems in terms of causality, diagnosis and management for clinicians and researchers alike [1]. The myriad symptoms of the syndrome also present major diagnostic problems for primary healthcare providers. Unfortunately, lack of specialised knowledge (within the healthcare system) and scepticism on the part of some often leads to a breakdown in trust and confidence between patient and physician. This problem was highlighted in an investigation of perceptions in patients with CFS who had been referred to a specialised clinic [2]. 68 patients completed a survey assessing their satisfaction with the medical care offered at the clinic. Two-thirds of the sample expressed feeling dissatisfied with the quality of care received during their illness. Furthermore, these patients were more likely to describe delay, dispute or confusion over diagnosis. Many of these same patients had received a psychiatric diagnosis for their symptoms which they rejected. In addition, this sub-group of patients perceived doctors as dismissive, sceptical or lacking in knowledge of CFS and felt that advice given was inadequate or conflicting. In contrast, satisfied patients believed doctors to be sympathetic and supportive of their condition. A major conclusion drawn from this study was the importance of understanding and effective communication between doctor and patient in dealing with CFS. Patients, it would seem, preferred GPs who, although admitting a lack of knowledge on the subject, offered empathy and support.

There are many avenues open to sources of information on CFS for doctors and patients alike. Unfortunately, too often they offer inaccurate and conflicting advice. In an age of increasing access to the internet, an Australian survey [3] reviewed 225 websites over a two week period. Widely differing views were found from websites offering information with regard to treatment for CFS. There was, however, general agreement that graded exercise and avoidance of prolonged rest were the most successful management strategies for sufferers. 64% of the sites offering advice had a named author. However, only a quarter to a third of the sites reviewed advised readers to clarify the information proffered with an appropriate health physician or avoided the inclusion of inaccurate statements. The report concluded that physicians should provide guidance for patients as to which internet sites to trust. It also recommended that GPs should be made fully aware of the nature of the information being accessed by patients. Misinformation leading to possible distress for the patient could then be avoided.

The strategies, employed by physicians in Sweden, to categorise, diagnose and treat CFS and fibromyalgia (FM) patients was investigated by Åsbring and Närvänen in 2003 [4]. During their study twenty-six physicians, all of whom had knowledge of working with CFS or FM, completed a semi-structured interview. Results from the study suggested that there was a discrepancy between the ideal role that the physician wished to fulfil and the reality of everyday work involving interaction with CFS patients. The physicians were concerned that their lack of specialist knowledge prevented them from providing proper healthcare support for their patients. This, the authors' concluded, led to the professional role being questioned by the patient. The point was also raised that some physicians were viewing CFS as a less serious illness than those conditions deemed to have 'disease status'. Further to this, scepticism was expressed on the part of the physicians as to the actual existence of CFS. Indeed, further studies have supported these findings [5]. It can be inferred from these studies, therefore, that there is continued diversity within the primary healthcare setting. Views ranging from questioning the existence of the syndrome to differing modes of diagnosis and management have been recently reported [5]. As an example of this, the Department of Public Health and Primary Care at the University of Hull conducted a national survey of General Practitioners (GP) and their beliefs regarding CFS [6]. Their research produced a comprehensive report on the current state of affairs within the United Kingdom. 300 questionnaires were sent out to GP surgeries in ten Regional Health Authorities in England, Scotland and Wales. Five of the Authorities surveyed had specialised centres for CFS, five did not. The five regions that did not have specialised centres for CFS were matched as closely as possible to those that did. One conclusion from the study indicated that although the GPs in the areas with specialised CFS clinics were more likely to belief that the condition existed, there was no difference in their 'propensity to diagnose' than those in areas without specialised services. This suggests that although GPs in the areas with specialised centres were aware of the clinics' existence, there was limited flow of specialist knowledge from the centres to primary care.

GPs' perceptions of patients with CFS have been studied in comparison with other syndromes. In 2004, Raine et al. [7] compared GP beliefs regarding patients with CFS to those with Irritable Bowel Syndrome (IBS). Their findings indicated that the attitudes of GPs to either CFS or IBS dictated subsequent management of the illness. The research concludes that these perceptions would ideally need to change to facilitate successful treatment implementation.

The aetiology, diagnosis, management and treatment for patients with CFS remain unclear and the need for further research into this condition is vital. This paper does in some cases set out to replicate the findings of other studies but was conducted as part of a wider research project investigating healthcare evaluation and patient education in CFS.

### Objectives of the present study

The aim of the current study was to investigate the opinions of CFS sufferers themselves, regarding diagnosis and treatment, and compare them to the current thinking of GPs from a different Health Authority but within the same geographical region. We could then address the question of whether the situation had changed in the light of two important reports being in the public domain, namely those by the Royal Colleges [8] and the National Task Force on CFS/ME [1]. Information from the study could then be used to develop educational literature for both GPs and patients regarding CFS diagnosis and management.

## Methods

Ethical approval for this study was granted by the Gwent Health Authority. All data were coded to ensure the anonymity of both the patients and the GP Surgeries taking part.

### Design

The study took the form of a simple survey proforma and the questions designed as a preliminary point of reference for the production of educational literature for patient and GPs.

### Participants

#### Patient sample

Patient recruitment was from an existing research panel. All of these volunteers had been diagnosed using the Centre for Disease Control (CDC) criteria for CFS [9] at a specialised outpatient clinic some years previously. 197 CFS sufferers were surveyed by postal questionnaire.

#### Primary care sample

120 questionnaire booklets were distributed by members of staff at the Gwent Health Authority Headquarters into the official postbags for the area's GP practices.

Neither the GPs nor patients were sent reminders to return the booklets following the first mail shot.

### Procedures

#### Questionnaires

Two short booklets were compiled, by the authors, to glean us much comparable data between the patients and GPs as possible. The booklets complied for the GPs were done so in as concise a manner as possible in order to maximise response rates in a profession where time is limited. Patient booklets elicited similar information in order to establish comparability with the GP sample. However, patients were also required to comment on any therapy they might have received and their current state of health. In this way it was hoped that data collected from the research panel regarding past diagnosis and management could be compared to the up-to-date information given by the GPs. In this way we would be collecting data relative to our research based on work from previous studies [6].

##### Patient questionnaires

First and most importantly, the patients were asked if they were still suffering from CFS, and for how long the condition had presented itself. The questionnaire then went on to illicit information regarding the level of primary healthcare received. This included the diagnostic tests and treatment options offered. Patients were also asked if any of the management/treatments offered were successful and asked to rate their health status using a previously validated current state of health measure [10]. This measure assesses the severity of their illness on a 5–item scale ranging from 'worse than at any stage' to 'almost completely recovered'.

##### GP questionnaire

The GPs surveyed were asked two fundamental questions: (a) did they believed that there was a single entity called Chronic Fatigue Syndrome (often known as Myalgic Encephalomyelitis), and, if so, (b) had they ever diagnosed patients with this illness. If the respondent answered 'no' to both of the above questions they were asked to return the survey. GPs who answered 'yes' were then asked to supply details of diagnostic criteria [9,11] and management regimes offered to their patients. They were also asked if their surgery carried any information booklets for patients and if so their source.

Both patients and GPs were asked if they would be prepared to comment on literature complied by healthcare professionals in the future.

### Data analysis

Descriptive statistics were performed on the categorical and continuous data. Open-ended questions were collated and categorised.

## Results

92 patient questionnaires were completed and returned giving a 48% response rate. A further 21 were returned to sender leaving 84 unaccounted for. Of the questionnaires distributed to the GPs, 45 were returned, two of which were blank giving a 39% response rate.

### Patient survey

Of the 92 patient respondents, 78 reported that they were still suffering from CFS (84.8%), indicating a 15.2% recovery rate for the sample. The mean illness duration for the group was 13.14 years (range = 3 to 32 years, s.e.m = 0.63). When asked to rate their current state of health, 2.2% of the sample reported feeling 'worse than at any stage' of their illness, 16.3% reported feeling 'bad', 32.6% were feeling 'bad with some recovery', and 33.7% were 'recovering with occasional relapses'.

51.6% of the sample indicated that their GP had diagnosed their condition, taking on average 6.58 (range = 2 to 20 appointments, s.e.m = 0.78) appointments to do so. When asked if the patient believed that they were suffering from CFS before their GP's diagnosis, 52.6% stated that this was the case. The patients were then asked to whom they had turned to for information on CFS (other than their GPs). The majority of respondents (52.2%) had contacted the ME Association. However, 63% stated that they had gained information form 'other sources'. These included 'friends or colleagues with CFS' (17.2%) and newspaper or magazine articles (62.1%).

The patient sample was then questioned about diagnosis and management. 82.6% of those surveyed reported that their GP had conducted investigative tests to exclude other diseases. These tests included: (a) a full range of blood tests (90.9%), (b) a test for the Epstein-Barr virus (51.9%), (c) tests for other viral infections (48.1%), and (d) 'other' tests (18.2%). Those described as 'other tests' included thyroid function tests (n = 4), hormone function tests (n = 3), rheumatoid factor (n = 3), ECG (n = 1), lung function (n = 1) and MRI scan (n = 1).

In terms of CFS management, 59.8% of the patients had been offered treatment by their GP, the most popular being antidepressant therapy (92.7%) or analgesics (not including 'over the counter' medicines) (36.4%). Of the 51 patients who had taken antidepressant medication, 18 reported that this form of therapy had made their symptoms worse, twenty-two reported no change in their symptoms, nine said that the therapy had improved their symptoms and one patient reported that antidepressant therapy had returned them to normal health. One patient did not respond to this follow-up question. Twenty patients reported that they had been prescribed pain relief to manage their symptoms. One reported that analgesics had made their symptoms worse; thirteen reported no change in their symptoms and six felt that the analgesic medication had made their symptoms better. Other management strategies offered by the GPs in the survey included Cognitive Behaviour Therapy (CBT), Grade Exercise Therapy (GET), Occupational Therapy (OT) and Counselling. 33 of the 92 respondents had also been referred to hospital consultants (other than the one who later confirmed the diagnosis of CFS). Table1 lists the outpatient departments attended by the survey responders before being referred to the specialised clinic.

**Table 1:** Outpatients departments attended by CFS sufferers before attending a dedicated CFS clinic.

**Outpatient Department**	**Number of Attendees**
General Medicine	16
Cardio/Thoracic	3
Psychological Medicine	3
Immunology	2
Dentistry	1
ENT	1
Neurology	1
Virology	1

In addition to these data two patients attended Homeopathy clinics and another received a private consultation. The remaining two patients did not offer a response to the question. 77.2% of the survey respondents had tried 'alternative' therapies to alleviate their symptoms spending on average £981. One respondent reported spending as much as £7000 on alternative therapies.

Finally, 89% of the patients sampled said that they would be willing to comment on information booklets aimed at CFS diagnosis and treatment.

### GP survey

Of the 45 GP respondents, 55.8% believed that the condition called CFS existed and 67.4% of these had diagnosed patients with CFS. On average, 6.2 (s.e.m = 0.97) separate appointments were required to diagnose the condition. None of the GPs who completed the survey used the CDC or Oxford criteria for CFS, preferring to either conduct investigative tests to rule out other illness (68.8%) and/or refer the patient on to tertiary care (65.6%).

When considering the sub-group of GPs who reported diagnosing CFS, 89.3% offered treatment strategies to the patient. None of the GP surgeries had trained nurses, occupational therapists or physiotherapists capable of offering support, advice or treatment to sufferers in the primary care setting. They also reported being unaware if any such services were presently available in their locality. The most common form of treatment offered by the GPs who responded to the survey was antidepressant therapy. 84% of GPs prescribed Selective Serotonin Re-uptake Inhibitors (SSRI) antidepressants, 28% preferring Serotonin/Noradrenalin Re-uptake Inhibitors (SNRI) and 24% prescribing the Tricyclic and related antidepressants.

Only 14.8% of the surgeries surveyed carried information leaflets on CFS. Most of the literature, it was reported, was supplied by the ME Association.

In terms of referrals to tertiary care, 56.7% of the GPs surveyed were aware that there was a consultant in the area who specialised in CFS. 16.7% referred patients to General Medical out-patients clinics, 6.7% to Rheumatology clinics, 6.7% to Neurology clinics and 6.7% referred patients to Psychological Medicine.

54.5% of the GP respondents were prepared to answer a more detailed questionnaire at a future date and 42.4% were willing to comment on the information leaflets referred to previously.

## Discussion

This paper aims to describe the current thinking of GPs from a single health authority in Wales. The data was collected as part of an ongoing project which included, amongst others, the need highlight whether GPs were being made aware of up-to-date information on CFS centres of excellence, its diagnosis and management. If not, our aim was to rectify this by offering to provide GP surgeries with information compiled by healthcare professionals in the field of CFS research.

It is acknowledged that the response rates, by both patients and GPs, for the current survey may appear to be low. However, a recent survey of members of local ME groups (supported by Action for ME and the ME Association) recorded patient response rates of 47% [12]. Furthermore, a ten-centre survey [5] reported GP response rates ranging from 35% to 55%. The latter is in sharp contrast to data presented recently by Bowen et al. [13] indicating a 77% GP response rate to their CFS survey. The data from this survey, however, was collected from GP surgeries served by medical laboratories within their region which may have acted as an incentive to respond. In addition to the differences in the method of sampling, data indicating initial response rates are not recorded; only those from the post-follow-up. With this in mind, the response rates of 48% for patients and 39% for the GPs in the present study seem more indicative of the types of group sampled. We can, therefore, put forward the view that the data reported here does represent an accurate portrayal of patient and GP opinions as long as it is discussed in relation to the situation within Wales and not to the UK as a whole. To further support this, data from the patient research panel group includes respondents who have recovered from Chronic Fatigue Syndrome (CFS). The current state of health measure also indicates that the health status of the group follows a similar profile to that of patients from previous studies (Thomas and Smith, in preparation). Likewise, the GP respondents are split approximately fifty-fifty between those who believe that the condition called CFS exists and those who do not. Therefore, no bias on the basis of patient 'wellness' or GP 'belief' in CFS is indicated here.

Scepticism on the part of GPs in recognising that CFS actually exists remains a problem to this day. Only 56% of the GP responders believe that CFS is a recognised condition despite findings from reports by the joint Royal Colleges and the National Task Force being in the public domain. Of the 44% who did believe that the illness exists, none reported using the CDC or Oxford criteria for CFS definition. This is surprising as both case definitions are readily available to medical and research staff and patient groups alike.

When questioned, only 57% of the GPs surveyed were aware that a CFS specialist was consulting within their local health authority region. The majority of those who were not aware of this referred patients to general medical outpatient clinics. This has been problematical in the past. Unless the patient is fortunate enough to be referred to a physician who, if not knowledgeable on the subject, is aware of specialist help, this will invariably result in the patient being told that there is 'nothing physically wrong with them'. The patient then returns to a GP who has two courses of action open to them: refer the patient to another outpatient department or try to manage the patient's condition themselves. This is bound to result in frustration on the part of the physician, who has the patient's best welfare at heart, as much as the patient.

Comparisons between the patients who had received a diagnosis from their GP and the GPs, who reported diagnosing CFS, both indicate that the process took approximately 6 appointments. Interestingly, the range of 2 to 20 appointments to diagnose the condition is identical for both groups. It is important during the process of diagnosing CFS, that other illnesses presenting fatigue-like symptoms are ruled out. However, only two-thirds of the GP respondents reported conducting further investigations to exclude these conditions.

It is encouraging to note that more GPs are currently offering treatment strategies compared to the past (89% and 60% respectively). However, antidepressants remain the preferred mode of treatment. Antidepressant therapy does have its role to play in treatment strategies in certain circumstances as described previously (Thomas and Smith, in preparation). But reports by CFS patients of heightened sensitivity to such medication have been widely documented and antidepressants should be prescribed with caution. In addition, findings from successful treatment trials of CBT and GET for the treatment of CFS do not seem to have filtered through to primary healthcare.

The authors acknowledge that General Practitioners' time and resources are limited and that being able to keep up with advances in research is a luxury they can ill-afford. Following the report to the Chief Medical Officer, the Medical Research Council recently set aside a considerable sum of money to support CFS research projects and subsequent information dissemination within the UK. Unfortunately, none of the funding found its way to projects in Wales. This means that the Principality currently trails behind the rest of the country in terms of resources available for research in this area. Due to this short-fall in Welsh funding, it is not surprising that the GPs represented in our survey lack confidence when dealing with patients with CFS.

On a positive note, almost half of the GPs surveyed would welcome helpful, practical advice written by healthcare professionals when dealing with patients whom they suspect may have CFS. However, the state of affairs with regard to the past experiences of the research panel patients and the current opinions of the GP respondents is all too familiar.

## Conclusion

The proposed next step is to produce informative material for both GPs and patients. This material needs to be compiled in conjunction with CFS specialists and will include details of centres of excellence, diagnosis and management.

## Abbreviations

CBT – Cognitive Behaviour Therapy

GET – Graded Exercise Therapy

CFS – Chronic Fatigue Syndrome

CDC – Centre for Disease Control and Prevention

GP – General Practitioner

IBS – Irritable Bowel Syndrome

OT – Occupational Therapy

SNRI – Serotonin/Noradrenalin Re-uptake Inhibitor

SSRI – Selective Serotonin Re-uptake Inhibitors

## Competing interests

The author(s) declare that they have no competing interest.

## Authors' contributions

MAT study design, recruitment of participants, collation of databases, data analysis and manuscript preparation. APS advice on study design and manuscript.

## Pre-publication history

The pre-publication history for this paper can be accessed here:


